# Bilateral Congenital Preauricular Fistula of the Cavum Conchae

**DOI:** 10.7759/cureus.33329

**Published:** 2023-01-03

**Authors:** Shotaro Koizumi, Takuya Yoshida, Kento Ishigaki, Ryoukichi Ikeda, Jun Suzuki

**Affiliations:** 1 Otolaryngology-Head and Neck Surgery, Tohoku University School of Medicine, Sendai, JPN; 2 Otolaryngology-Head and Neck Surgery, Iwate Medical University School of Medicine, Yahaba, JPN

**Keywords:** ear surgery, auricular infection, sinectomy, cartilage, external auditory canal

## Abstract

A 16-year-old female who had left auricular sinus infections was admitted to our hospital. On physical examination, bilateral sinus tract openings were noted at the cavum conchae. We used a surgical microscope to complete the total resection of the bilateral sinus at the cavum conchae. Also dissected was the cartilage from the cavum conchae. To our knowledge, surgical excision of cavum conchae sinuses has not been previously described.

## Introduction

The external ear deformity known as congenital preauricular sinus is frequent in children. Most patients have a skin pit in front of the ascending branch of the helix as their primary symptom [1]. No treatment is necessary when a patient has no obvious symptoms [2]. Infants and young children are typically the first to get a preauricular fistula infection, which necessitates surgery. However, because infants and young children are unable to comply, parents are obliged to help with dressing changes.

Although most preauricular sinuses are found anterior to the external auditory canal [3], a few have been found in unusual places such as the supra-auricular region, postauricular region, lobule, ascending helix crus, superoposterior edge of the helix, and tragus [4,5]. A previous study found that congenital sinuses at the cavum conchae are very rare [6]. Hiraide et al. reported 382 cases of congenital preauricular sinus, and only two cases (0.5%) were observed at the cavum conchae [6]. In addition, there has been no report regarding detailed surgical techniques. Herein, we report the unique case of bilateral congenital preauricular sinus at the cavum conchae and a novel surgical procedure.

## Case presentation

**Figure 1:**
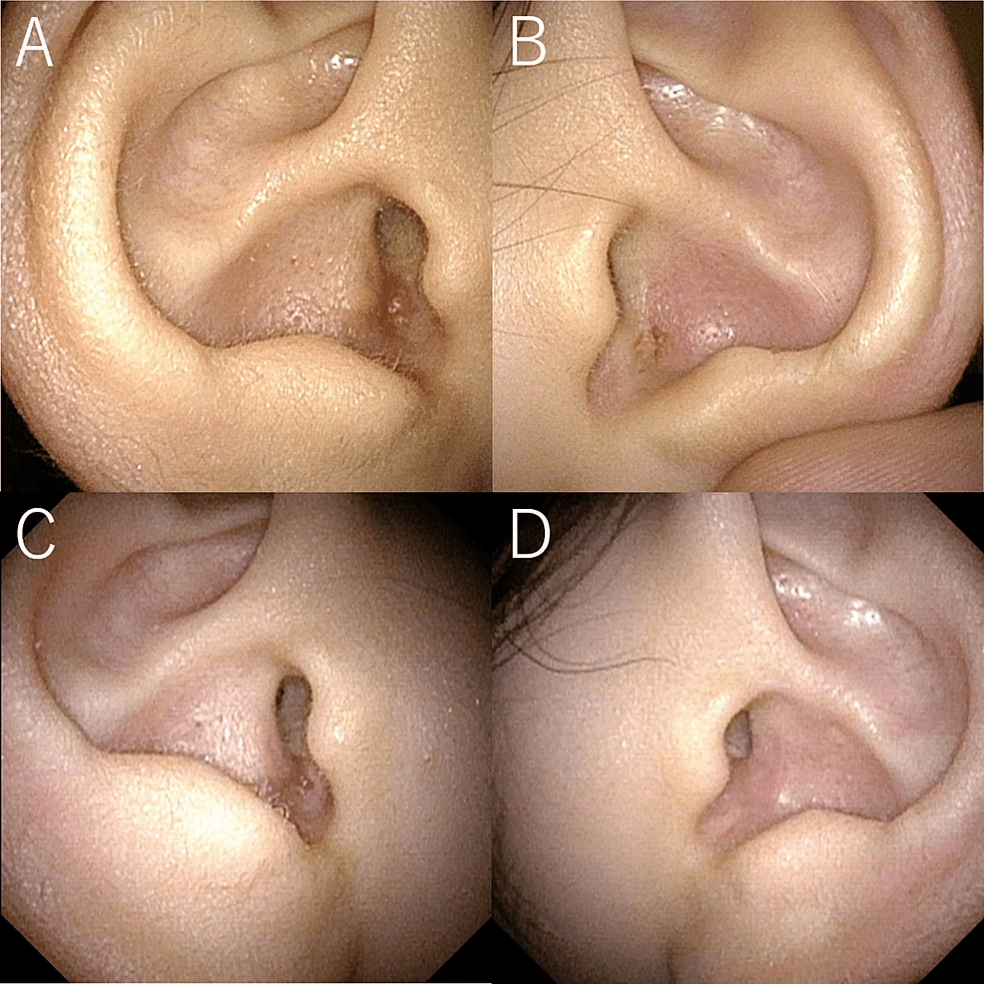
Right (A and C) and left (B and D) auricles before (A and B) and after (C and D) surgical treatment.

A 16-year-old female presented to our hospital with infections of the left auricular sinus four times in the past year. Physical examination revealed bilateral sinus at the cavum conchae (Figure 1A and Figure 1B).

**Figure 2:**
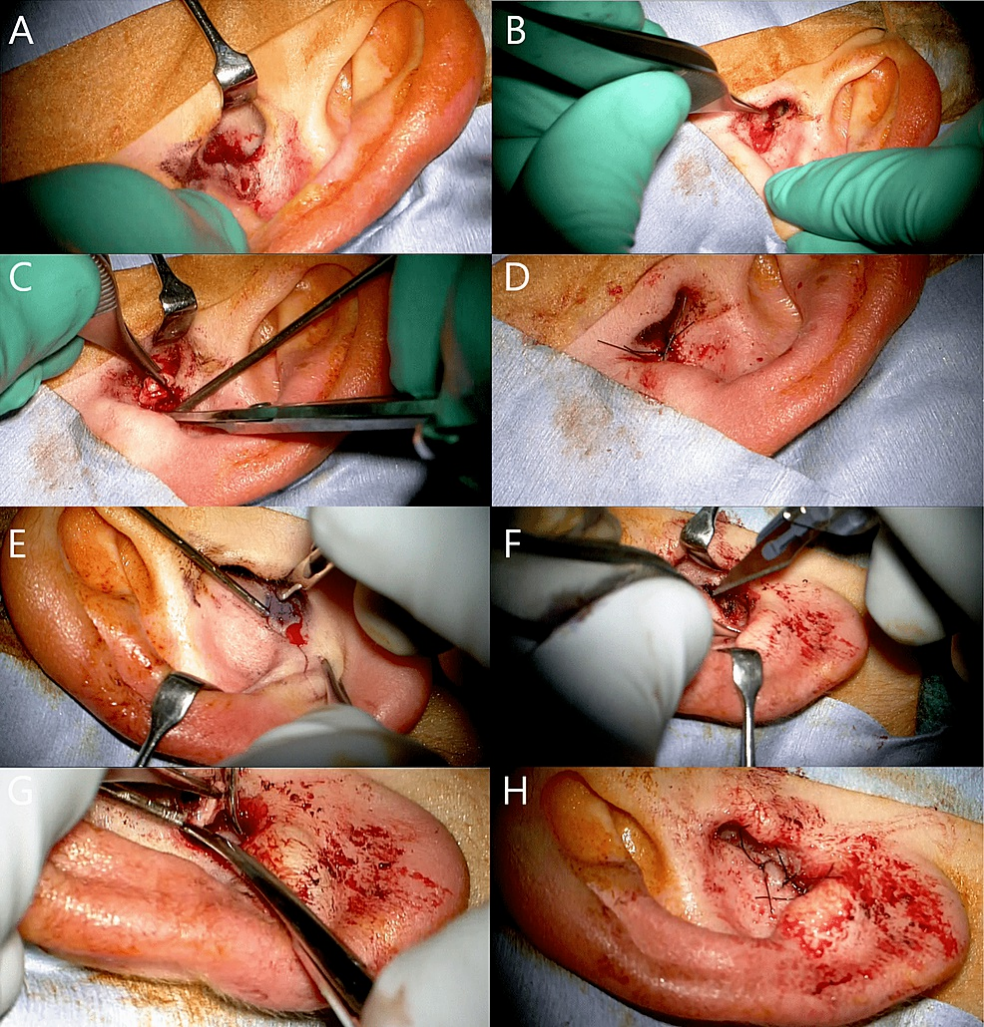
Surgical treatment of the left ear (A-D) and right ear (E-H). A: Skin incision. B: The sinus including the skin lesions were resected. C: The cartilage of the cavum conchae was dissected. D: Skin suture.

We performed total resection of the left sinus at the cavum conchae under local anesthesia using a surgical microscope. After the sinus tract was probed with a lacrimal probe and cannulated with Introcan Safety® 3 Closed IV Catheter 24G (B. Braun Melsungen AG, Melsungen, Germany), 2% pyoktanin blue injection (FUJIFILM Wako Pure Chemical Corporation, Osaka, Japan) was administered. Before making the incision, the location and depth of the track were confirmed with the lacrimal probe placed into the preauricular sinus pit. The depth of the track was 1.5 cm. After performing an elliptical incision around the hole (Figure 2A) and separating the surrounding tissues, the auricle cartilage could be seen (Figure 2B). The cartilage of the cavum conchae was also dissected (Figure 2C). The surgical lesion was irrigated with a normal saline solution. The surgical wound was sutured using 5-0 ETHILON (ETHICON, Inc., Raritan, NJ, USA) (Figure 2D).

**Figure 3:**
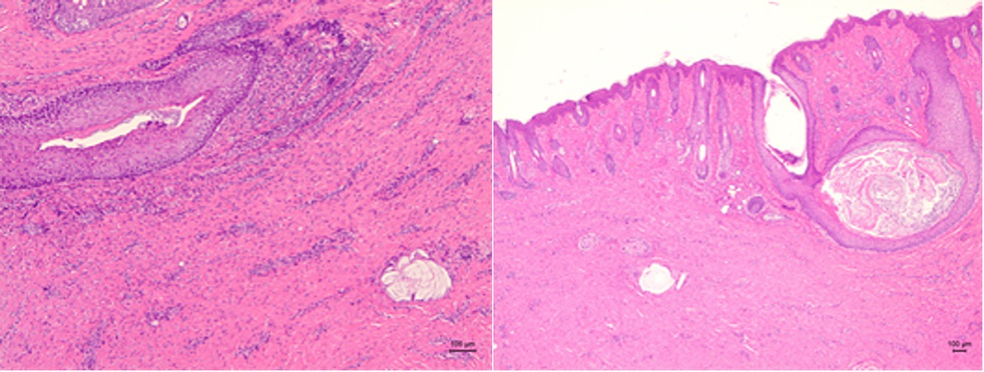
Histopathological study. The fistula connected to the epidermis was covered with keratinized stratified squamous epithelium. Inflammatory cells were infiltrating the fistula.

Histopathological examination revealed that the fistula was covered with keratinized stratified squamous epithelium that was continuous with the epidermis. A stratified squamous epithelium that contained keratinized material and expanded like a mass covered a portion of the fistula. Keratophagocytic multinucleated giant cells, histiocytes, and inflammatory cell infiltrates, which constituted a foreign body response to keratides, were present around the fistula (Figure 3). No evidence of a tumor or malignancy was observed.

Two months after surgery, she had a right auricular infection. Five months later, the same surgery was performed on the right ear as on the left ear under general anesthesia (Figure 2E-2H). Three months after the right ear surgery, the external auditory canal skin was epithelialized, and no external auditory canal stenosis was observed. No recurrence was found (Figure 1C and Figure 1D).

## Discussion

This is the first report of surgical resection of the variant type of preauricular sinus of the cavum conchae. Preauricular sinus has an estimated incidence of 0.1%-0.9% in the United States [7], 0.9% in England [8], 2.5% in Taiwan [9], 2.6% in Japan [10], 2.5% in Korea [11], and %4-10% in some African countries [7]. Choi et al. classified a congenital fistula at the ascending helix crus with tracts running posterior or posteroinferior as a “variant of the preauricular sinus” [4]. The variant type of preauricular sinus has been reported in several studies. Kim et al. reported that the variant group was 9.4% of whole subjects who underwent congenital periauricular sinus excision [12]. The variant type of fistula was observed in the ascending helix crus, infra-auricular area, supra-auricular area, and anterior to the tragus. Hiraide et al. also reported 382 cases diagnosed during school examinations. Less common variant types include the cavum conchae (0.5%), cymba conchae (1%), posterior helix (4.7%), anthelix (0.8%), incisura terminalis (0.8%), crura of the anthelix (1.3%), and posterior auricular area (1.9%) [6].

There has been no report regarding detailed surgical techniques for the variant of the preauricular sinus at the cavum conchae. To prevent a recurrence, complete excision is recommended when surgical excision is necessary. Different excision methods, such as sinectomy and supra-auricular approach, have been described [13]. In the standard simple sinectomy, the skin around the pit is removed in an elliptical shape, while the individual sinus tract is followed to its end [14]. Technical alterations have been discussed, either with or without the excision of a small piece of cartilage from the ascending helix of the limb. Other techniques, such as the supra-auricular approach, use an extended incision to cut through soft tissues, including the sinus tract, using the topographical tract boundaries rather than the real preauricular sinus tract. A systematic review of the surgical outcome of preauricular sinus showed that sinectomy using the microscope resulted in the lowest sinectomy recurrence rate (1.9%) [13]. We performed a simple sinectomy and partially resected the cartilage of the cavum conchae. Removing a small portion of auricular cartilage along with the sinus tract has been controversial [15]. The average sino-cartilaginous distance was 472 μm. The sino-cartilaginous distance was less than 0.5 mm in more than 50% of specimens. In almost all of these, the epithelial tract was in continuity with stromal tissue histologically identical to the perichondrium [15]. They concluded that most sinus tracts might be challenging to separate from the cartilage, according to sino-cartilaginous distances. Regular removal of the sinus tract and a small part of the auricular cartilage may result in a more thorough excision and aid in preventing recurrence. If a sinus is present in the cavum conchae, the removal of the sinus exposes cartilage, which requires either a concomitant resection or reconstruction with a skin flap. Clinical doctors should be aware of the anatomical variations in facial nerve course to avoid an injury [16,17]. In our case, the defect was not reconstructed but sutured. Although stenosis at the entrance of the external auditory canal is a concern, no significant stenosis was seen in this case, and this method was thought to be helpful.

## Conclusions

In this study, we reported the case of bilateral congenital preauricular sinus at the cavum conchae. A simple sinectomy and partially resecting the cartilage of the cavum conchae could be useful in these cases.
